# Effects of seasonal chronic heat stress on body thermoregulation, cortisol release and uterine health in postpartum native Alentejana and Mertolenga beef cattle

**DOI:** 10.1186/s12917-025-04810-z

**Published:** 2025-06-05

**Authors:** Luís G. Capela, Inês C. Leites, Luísa M. Mateus, Ricardo P. Romão, Rosa MLN. Pereira, Luís Lopes-da-Costa

**Affiliations:** 1 Biotechnology and Genetic Resources Unit, National Institute of Agrarian and Veterinarian Research (INIAV), Santarém, 2005-048 Portugal; 2 Center for Interdisciplinary Research in Animal Health (CIISA), Faculty of Veterinary Medicine, University of Lisbon, Lisbon, 1300-477 Portugal; 3 Associate Laboratory for Animal and Veterinary Sciences (AL4 AnimalS), Lisbon, 1300-477 Portugal; 4 Environment and Development (MED), Mediterranean Institute for Agriculture, University of Évora, Évora, 7006-554 Portugal

**Keywords:** Bovine, Hair Cortisol, Heat Stress, Metabolic Profile, Thermoregulation, Uterine Health

## Abstract

Selection towards resilience to heat is a main goal to mitigate Heat Stress (HS) effects. Resilient breeds entail a genetic pool to uncover novel thermoregulatory mechanisms. This study evaluated the response to seasonal chronic HS and mechanisms behind thermo-resistance in two heat-resilient native cattle breeds. Multiparous cows of *Bos taurus* native breeds Alentejana (ALT, *n* = 34) and Mertolenga (MERT, n = 55), maintained in continuous pasture, were examined at 40 ± 6 d postpartum at the end of Summer (S) and Winter (W). Cow-side temperature-humidity index (THI) and body temperatures were measured, blood was collected for BHB, leptin, total T3 and T4, neck hair was collected for cortisol measurement, and the genital tract was evaluated by ultrasonography, endometrial cytology and biopsy. In ALT cows, rectal temperature was similar in both seasons, whereas in MERT cows it was greater (*p* < 0.01) in S compared to W. Mean THI of previous 60 days attenuated the increment in body temperatures, revealing a significant (*p* < 0.01) acclimation mechanism, which was more efficient in ALT. In summer, T3 decrease was more accentuated in ALT than MERT cows (34% vs. 13%, *p* < 0.05). This highlights the breed's specific thermoregulatory traits. Hair cortisol concentrations were not affected by season, thereby failing as a reliable indicator of HS in native breeds. Endometrial cytology proportion of polymorphonuclear neutrophils (PMN) was reduced with increasing THI (*p* < 0.01), independent of cortisol secretion. In conclusion, this study identified chronic stress phenotypic markers of beneficial thermoregulatory mechanisms of heat resilient cattle breeds, relevant for selection programs.

**Supplementary Information:**

The online version contains supplementary material available at 10.1186/s12917-025-04810-z.

## Background

Climate change has increased the number, duration and intensity of heat waves in the Mediterranean basin [1]. This has increased the number and magnitude of heat stress (HS) episodes in beef cattle reared in continuous pasture systems, where direct sun exposure aggravates the HS effect [2–4], making it a serious constraint to animal welfare, fertility and productivity. Alentejana and Mertolenga breeds share the same region of origin. Some authors argue that they are sub-breeds of the historic Transtagana breed, which also includes the almost extinct Algarvia breed [5], their ancestor being the *Bos primigenius*. However, Mertolenga breed has physical characteristics that show a cross between *Bos primigenius* and *Bos desertorum* (desert ox) [6], leading to hypothetical differences in their thermoregulatory mechanisms and their efficiency. With a live weight of 600 kg for females and 1000 kg for males, Alentejana was used as draft cows in the past. The Mertolenga breed is smaller with a live weight of 450 kg for females and 800 kg for males [6, 7]. Regarding coat colour, Alentejana present a full brown color while Mertolenga range from white to full brown, this being a regional difference inside Mertolenga breed [6]. Both breeds evolved in the hottest and most arid region of Portugal having, in natural conditions, the first calving at 24–36 months [6–8]

In this scenario of seasonal chronic HS (at least mid-June to mid-September), selection of thermotolerant animals has emerged as a cornerstone towards the mitigation of deleterious HS effects, requiring novel knowledge on the adaptive physiological mechanisms to HS and beneficial phenotypic biomarkers [9].

Cattle, like homeothermic animals, regulate body temperature in response to environmental heat challenge through cooling mechanisms. One mechanism consists in latent heat loss through tachypnea and perspiration, cutaneous evaporation representing up to 85% of the latent heat loss of an adult cow [10]. Vasodilation of superficial vessels allows heat loss, a mechanism regulated by the sympathetic system [11]. A second mechanism consists in the regulation of internal thermogenesis through the control of metabolic rate. Thyroid hormones play a key role in this control, adjusting heat loss to heat gain [12], it being well-known that an increment in environmental temperature leads to a decrease in T3 [13] and T4 [14] blood concentrations. This is well documented in *Bos indicus* [15], and is a cornerstone mechanism in breed adaptability to HS [16, 17]. In contrast, in dairy cows, the increased metabolic demand for milk production does not allow a robust decrease in thyroid hormones, making these animals more susceptible to HS [18]. Although starvation also decreases thyroid hormone concentrations, this is concurrent with an increase in concentrations of non-esterified fatty acids (NEFA) and beta-hydroxybutyrate (BHB), associated to mobilization of fat reserves by lipolysis [19, 20]. Leptin also regulates the metabolic rate through food intake [21], with concentrations increasing in HS, reducing food intake [22], and decreasing negative energy balance, stimulating food intake [21]. Ultimately, efficiency in the above mechanisms will reflect the adaptive plasticity of the animal’s homeostasis and determine sensitivity to HS.

Plasma cortisol concentrations are hallmarks of stress, including HS, in livestock [23, 24], however they also reflect short, brief and non-recurrent moments of stress, for instance induced by handling. In contrast, hair cortisol concentrations (HCC) enable the quantification of cattle chronic stress over months, according to hair grow rate [25, 26], not masked by momentary episodes, as demonstrated in calves [27]. Therefore, HCC allow a robust picture of cortisol influence during an extended period of time, such as the hot season. Circulating cortisol concentrations affect cow reproductive physiology and fertility. High concentrations decrease estradiol release, depress estrus behavior [28], induce silent ovulations, and cause ovulation failure [29]. Dairy cows with lower postpartum cortisol concentrations became pregnant earlier compared to cows with greater concentrations [30]. Postpartum primiparous dairy cows with high plasma cortisol concentrations had an increased risk to develop metritis [31], and multiparous postpartum dairy cows with clinical metritis had greater HCC than healthy cows [32]. Neutrophils are the main cellular defense component in cows during postpartum resolution [33], but increased cortisol concentrations impair neutrophil lifespan, diapedesis, chemiotaxis and phagocytosis ability [34, 35].Cortisol release induced by HS may therefore be one concurrent cause for the poor reproductive efficiency and increased incidence of postpartum uterine disease of cows under HS. In fact, HS affected dairy cows show a greater incidence of postpartum uterine disease [36, 37].

In contrast to thermosensitive cattle breeds, native Alentejana and Mertolenga breeds (*Bos taurus*) reared under the traditional continuous pasture system of the south of Portugal maintain health and reproductive efficiency under acute or seasonal chronic HS. However, the physiological mechanisms behind this thermotolerance are unknown [38, 39]. Deciphering these mechanisms will allow the identification of biomarkers and strategies for the selection of HS resilient animals.

This study had two objectives: 1) evaluate the response to seasonal chronic HS and the mechanisms behind thermotolerance in native cattle; and 2) evaluate the relationship between HS, cortisol release, metabolic parameters and the postpartum uterine health status of cows.

## Methods

### Experimental procedures

#### Localization and meteorological data

This study was conducted in the Alentejo province of South Portugal, one of the hottest regions in the Iberian peninsula. In this region climate is defined by two well stablished seasons: Summer, which is dry and hot (average temperature of the hottest month > 22ºC); and winter, which is rainy and cold (average of the coldest month between 0 and 18ºC). All the herds are located in a Csa type Mediterranean climate according to Köppen-Geiger [8]. Retrospective meteorological data from the nearest weather station (< 3.5 km) was provided by IPMA (Portuguese Institute for Sea and Atmosphere, I. P.), and cow side data was registered with a hygro-thermometer (EXTECH-RH101, Rotterdam, Netherlands). Temperature and humidity data was used to calculate the Temperature Humidity Index (THI) according to the National Research Council [40] formula:

$$\text{THI }= (1.8 \times \text{ Tdb }+ 32) - (0.55 - 0.0055 \times \text{ RH}) \times (1.8 \times \text{ Tdb }- 26)$$

where, T_db_ = dry-bulb air temperature (°C) and RH = relative humidity.

THI60 was calculated as the mean THI value of the previous 60 days.

#### Animals and handling

The study was conducted between September 2020 and September 2022, using 5 herds distributed in 2 beef cattle farms (two Alentejana herds in one farm and three Mertolenga herds in another farm) owned by the respective breeds’ associations.

**Fig. 1:**
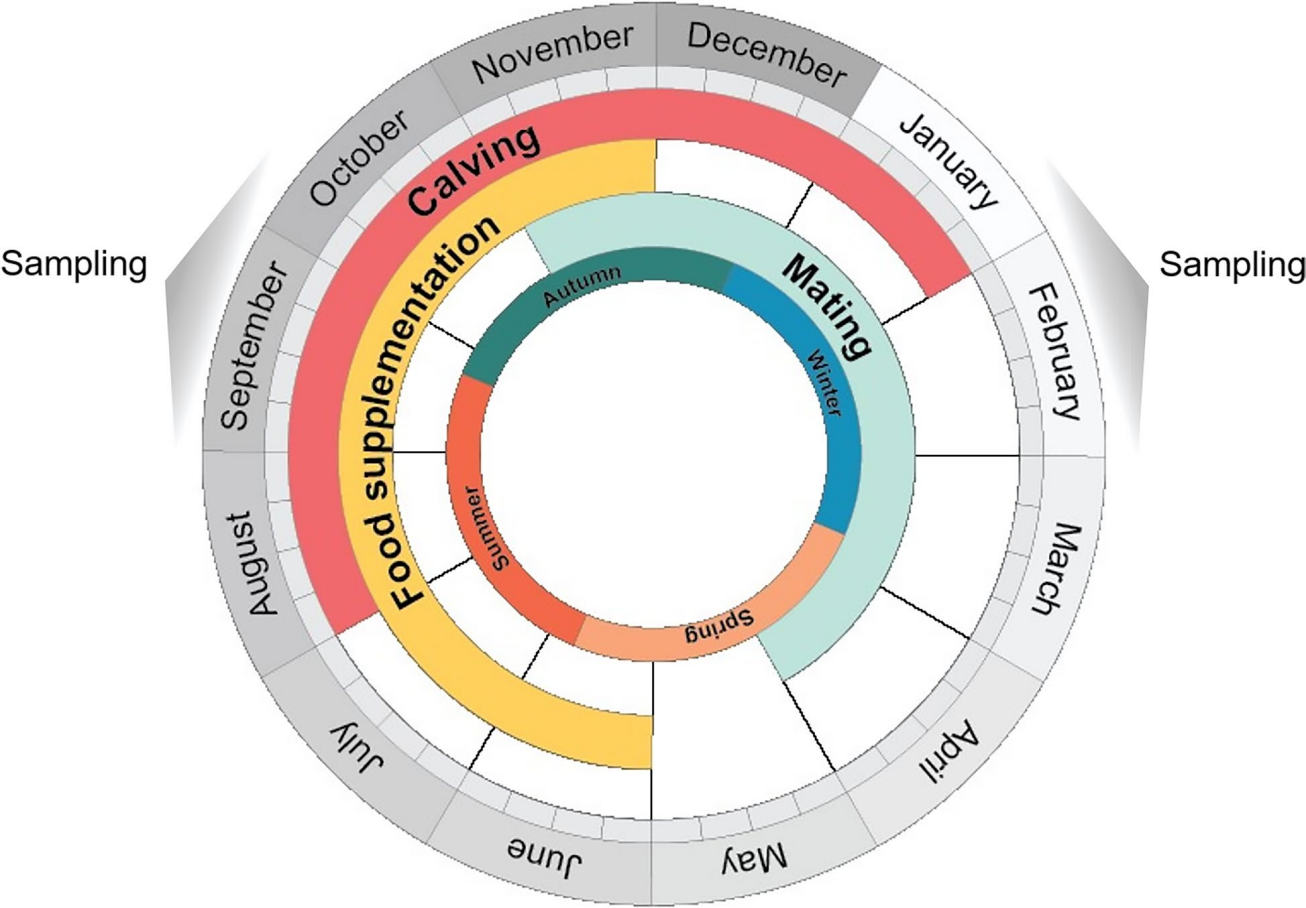
Graphical representation of herd management and sampling period of the study

The 5 herds had similar management, pursuing the traditional continuous pasture system. Cows graze on natural pasture (supplementary Table 1 A), under natural edaphoclimatic conditions, including natural shaded areas, with ad libitum access to water. Hay supplementation (supplementary Table 1B) was available ad libitum during seasonal pasture shortage (June to November). Prophylaxis include vaccination against clostridiums and deworming twice a year. Reproductive management consisted of a natural breeding season, excluding assisted reproductive techniques, from November 1 to April 30, by bulls of the respective breeds (ratio of 1:40), and a calving season from August to January. Herd management is illustrated in Fig. 1. Multiparous cows (*n* = 89) of Alentejana (AL, *n* = 34, age: 86.7 ± 26.7 months; parity: 3 ± 0.7) and Mertolenga (MERT, *n* = 55, age: 87 ± 40 months; parity: 3.0 ± 1.6) breeds were enrolled in the study following calvings in winter (W, *n* = 47) and summer (S, *n* = 42) as shown in Fig. 1 (S = July to September; W = January to March). Only Mertolenga cows with mostly brown coats were included. Cows with clinical disease, dystocia, retained placenta, as well as twin-calving cows were not enrolled in the study.

#### Ocular thermography and body temperatures

**Fig. 2:**
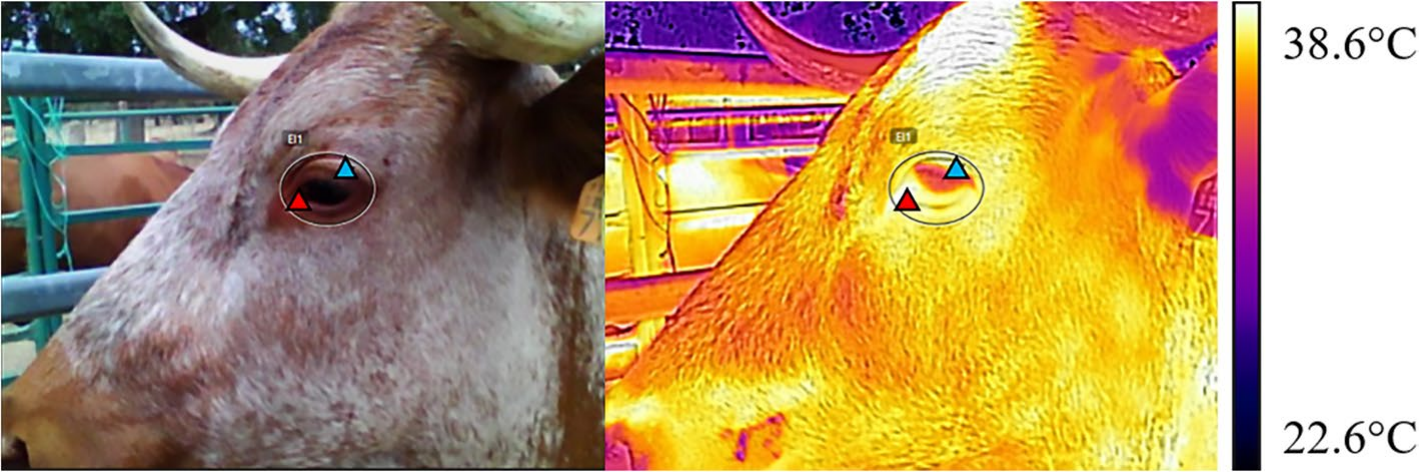
Digital (left) and thermographic image (right) of the same cow. Circle around the eye illustrate the place where the ocular measurements took place, red and blue triangles showing the maximum and minimum temperatures, respectively, calculated using FLIR Tools^TM^PC software

Ocular thermographs were acquired at 1 m from the cow, perpendicular to the left eyeball, with the cow placed in the shade to avoid artifacts caused by sun exposure, as described by [41]. Thermographs were taken with a FLIR®EX8 thermographer (Teledyne Flir, Oregon, USA), with an emissivity 0.98, and analyzed with software FLIR Tools^TM^PC at 76.800 pixel (320X240) resolution and 0.05 °C thermal sensitivity. For each photograph, a circle was drawn around the eyeball, including the skin of the eye cavity and the lacrimal gland (Fig. 2) in which the maximum and minimum temperatures were measured (OcularMax and OcularMin, respectively). Vaginal and rectal temperatures were acquired with a DIGI-VET SC12 thermometer (Kruuse®, Langeskov, Denmark) with an accuracy of 0.1 °C.

#### Blood metabolic parameters

Blood was centrifuged at 2000 × g for 15 min, serum aliquoted and stored at −80ºC. Serum BHB was measured using a handheld meter (Freestyle Precision NEO, Abbott®, Fremont, United States) with Optimum β-Ketone strips, as validated by [42]. Serum Leptin was measured with the Cusabio Bovine Leptin ELISA kit according to manufacturer (CSB-E06771b-96 T; Houston, USA). The intra-assay and inter-assay coefficients of variation were 2.8% and 1.9%, respectively. Total T3 and T4 concentrations were measured by chemiluminescence (IMMULITE 1000, Siemens) using LHT31 and LKT41 kits (DPC®, Los Angeles, United States), according to [43]. The intra-assay and inter-assay coefficients of variation were 3.3% and 2.1% for T3 and 5.3% and 3.0% for T4, respectively.

#### Hair cortisol assay

Hair was shaved with an electric clipper on the left side of the neck and close to the skin in an area of approximately 25 cm^2^. Only brown hair was sampled, as hair pigmentation influences cortisol concentrations [44, 45]. Since the hair grows approximately 0.6 mm per day [46], samples (2–3 cm) approximately reflect 60 days of cortisol incorporation.

Shaved hair was placed into a 2 mL Eppendorf and immediately stored in a cool place protected from light. Extraction was conducted as described by [27], with minor changes. Briefly, 200 mg of hair were placed into 15 mL conical tubes, washed with 3 mL 2-isopropanol (VWR, Radnor, USA), vortexed for 1 min and the supernatant discharged. This procedure was repeated three times or until the supernatant cleared, and samples were left to dry for 24 h at room temperature. Hair was then manually cut into fragments up to 2 mm, and 50 mg hair was weighed and placed in a glass tube within 1.5 mL methanol (VWR, Radnor, USA) to be extracted for 16 h in a stirring water bath (50 °C). Upon extraction, 0.75 mL was transferred into a 2 mL glass vial and evaporated under nitrogen stream at 50 °C. Finally, the dried extracts were reconstituted in 0.25 mL PBS at pH8. Cortisol concentrations were measured in duplicate using a commercially available ELISA Kit for Salivary Cortisol (DRG Instruments GmbH, Marburg, Germany) according to [47] and validated by [48], using a microplate reader (Fluostar Optima, BMG Labtech®), at 450 nm absorbance. Results were calculated using a 4PL curve fit. The intra-assay CV was 4.5%, and the inter-assay CV was 12.8% for the internal control, and respectively 12.8% and 13.6% for kit control 1 (low) and 2 (high) [49]. For recovery rate evaluation, a pool of hair samples was spiked with 1 ng/mL and 4 ng/mL of cortisol (Cerilliant® Sigma) and the procedure run in triplicate. Recovery rate was 96.4% for the pre-extraction and 97.9% for the post-extraction spiked samples.

#### Genital tract evaluation

Ultrasonography was undertaken with a portable ultrasound machine (ExaGo, IMV®, Bellshill, Scotland) fitted with a rectal linear 7.5 MHz probe. The type, number and size of ovarian structures, and the uterine texture and presence of intrauterine fluid were recorded. Samples for endometrial cytology were collected using an adapted cytobrush technique [50]. The brush was gently rolled along the length of two glass microscope slides, and the slides were labelled, air-dried and stained with a modified Wright-Giemsa® stain (Diff-Quick, MAIM SL, Barcelona, Spain). The percentage of polymorphonuclear neutrophils (PMN) was assessed from 400 cells (200 in each slide). A ≥ 5% PMN cut-off for cows with 40 DPP [51] was chosen for identification of cytological endometritis. Endometrial biopsies were collected in the previously gravid horn using a Kervokian–Younge endometrial biopsy instrument (Alcyon, Paris, France), under a low epidural anesthesia with Procaine (Pronestesic, Fatro, Bologna, Italy) according to [52, 53]. Briefly, the sterile biopsy instrument was introduced into the vagina inside a sanitary sheath, ruptured at cervical entry, and guided into the first third of the uterine horn, where a 1.0 cm^2^ endometrial sample was recovered. The sample was cut into two parts (for different analysis), one being immediately fixed in 4% paraformaldehyde, paraffin embedded within 24 h and stained with hematoxylin–eosin. The inflammatory infiltrate was blindly evaluated by a pathologist, being scored 0–3 (0, no inflammation; 3, severe inflammation), and cows that scored 2–3 considered with endometritis. The subsequent calving date was recorded and the intervals between calvings and calving to conception were retrospectively calculated.

### Experimental design and statistics

#### Experimental design

The sampling period occurred at the end of September (S) to allow the effect of chronic HS (90 days), and at the end of February (W), to allow full relief from previous HS effects. Cows were examined and sampled in the morning from 10 to 12 am at 40 ± 6 days postpartum (DPP), in a headlock system. Eye thermography, rectal and vaginal temperatures were immediately accomplished to avoid major changes caused by handling. Blood samples were then collected from the coccygeal vein with an 18G needle into 10 mL dry tubes for measurement of beta-hydroxibutirate (BHB), leptin, total T3 and T4 concentrations, and hair was shaved for cortisol measurement. Body condition Score (BCS) was assessed on a scale from 1 to 9 according to [54]. Gynecological ultrasonography allowed the identification and measurement of ovarian structures, the assessment of uterine involution. Subsequent endometrial cytology and uterine biopsy enabled the evaluation of endometrial immune cells and the presence of uterine inflammation.

For objective 1, the effects of Breed, Season and their interaction were evaluated in data from body temperatures, metabolic parameters and HCC, reflecting the breeds’ response to HS. For objective 2, the relationship between HCC, cow-side THI, THI60, body temperatures, metabolic parameters and uterine health indicators was evaluated.

#### Statistics

In this observational study, statistical analysis was performed using the SAS 9.4 version software (SAS 2024). The experimental unit considered was the cow. Categorical data were analyzed by Fisher’s exact test while parametric data was analyzed by applying ANOVA. After testing normal distribution by PROC UNIVARIATE the following variables were found to be normally distributed (Ocular temperatures, body temperatures, serum BHB, BCS, Leptin, T4, T3 and HCC) and non-normally distributed (uterine PMN). For objective 1, to test the fixed effects of Breed, Season and Breed*Season OcularMin, OcularMax, OcularMed, BCS, Vaginal, Rectal, T4, T3, BHB, Leptin, HCC and PMN, for normally distributed data, significant differences were determined using two-way ANOVA or Welch’s ANOVA when necessary, based on Levene’s test. For non-normally distributed data, the Kruskal–Wallis Test was used followed by Friedman test when necessary. Pearson correlations were calculated (PROC CORR) to investigate linear relationships. For objective 2, multiple regression analysis was conducted using PROC GLM and PROC GENMOD to assess effects and their interactions. The best fit model (goodness of fit and complexity) was evaluated using Akaike information criterion (AICC) and coefficient of determination (R^2^). All parameters were consecutively applied as dependent variables, while combinations of independent variables were used until the best fit model was found. Additionally, to visualize the effects of cow-side THI and THI60 on body temperatures, multiple regression graphs for each breed were performed on Origin2019 (Ogirinlab Corporation, USA) according to best fit models previously found on multiple regression analysis. Values were expressed as Mean ± SD, unless otherwise stated, and considered statistically different when p ≤ 0.05.

The fixed effects of Breed, Season and Breed*Season for all analyzed parameters and the significant GLM models are shown in Supplementary Table 2.

## Results

### Environmental temperature and THI

The recorded minimum and maximum temperatures were respectively −5.1 °C and 18 °C in W (THI = 23 and 63), and 8.7 °C and 43.8 °C in S (THI = 51 and 88), revealing the wide temperature range between seasons, characteristic of the climate type (in detail in Table 1). Mean cow-side environmental temperature and THI was greater (*p* < 0.0001) in S than in W (27.9 ± 5 °C, THI = 75 vs. 17.6 ± 4 °C, THI = 61). The mean THI of the previous 60 days (THI60) was greater (*p* < 0.0001) in S than in W (69.8 vs. 51.7). The cow-side THI and THI60 showed a positive correlation (r = 0.8, *p* < 0.0001), and were not affected by farm or herd.

**Table 1:** Seasonal meteorological data

Parameter	**Summer**			**Winter**		
	Global (24 h)	Hottest hours (11am-4 pm)	Night (9 pm-6am)	Global (24 h)	Hottest hours (11am-4 pm)	Night (9 pm-6am)
Maximal temperature (°C)	33.8 ± 1.3	33.8 ± 1.3	25.5 ± 1.6	15.5 ± 2.3	15.5 ± 2.3	11.5 ± 1.3
Minimal temperature (°C)	15.9 ± 0.6	25.8 ± 0.9	15.9 ± 0.5	6.6 ± 0.9	10.5 ± 2.0	6.8 ± 0.7
Mean temperature (°C)	24.3 ± 0.9	30.1 ± 1.2	19.5 ± 1.1	10.6 ± 1.5	13.5 ± 2.1	8.9 ± 1.0
HR%	58.2 ± 3.3	35.9 ± 2.3	74.7 ± 4.2	83.6 ± 3.7	71.8 ± 6.8	90.2 ± 3.9
Maximal THI	79.0 ± 1.4	78.5 ± 1.2	72.5 ± 1.9	59.2 ± 2.9	59.2 ± 3.0	53.2 ± 2.0
Minimal THI	60.1 ± 0.9	72.1 ± 1.0	60.3 ± 0.9	44.4 ± 1.4	51.5 ± 3.1	44.8 ± 1.2
Mean THI	70.2 ± 1.2	77.0 ± 2.2	65.3 ± 1.5	51.4 ± 2.2	56.3 ± 3.0	48.6 ± 1.5

### Body temperatures

**Fig. 3:**
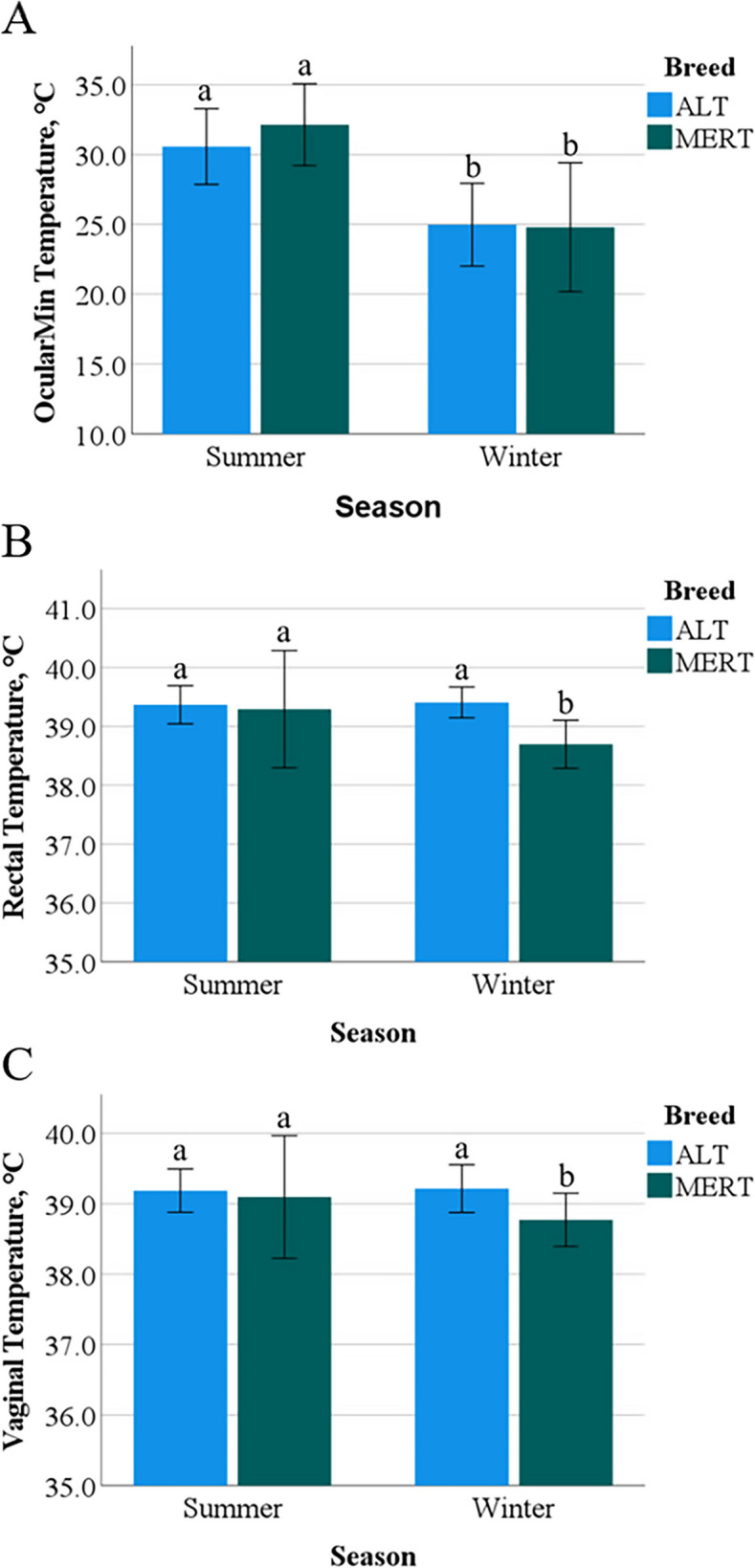
Interactions Breed*Season for OcularMin, rectal and vaginal temperatures in Alentejana (ALT) and Mertolenga (MERT) cows. **A** – ab, *p* < 0.0001; **B** – ab, *p* < 0.001; **C** – ab, *p* < 0.005

Cow-side THI showed a positive (*p* < 0.0001) correlation with OcularMin (r = 0.8) and OcularMax (r = 0.7) temperatures. For this reason, OcularMin was chosen for further analysis. OcularMin temperature was only affected by Season (*p* < 0.0001), being greater in S than in W (Fig. 3A). Rectal and vaginal temperatures, although affected by Breed (*p* < 0.01 and *p* < 0.05, respectively) and Season (*p* < 0.001), were mainly affected by the interaction Breed*Season (*p* < 0.001 and p < 0.01, respectively) (Figs. 3B and C). In fact, ALT cows had similar mean rectal and vaginal temperatures in S and W (*p* = 0.7), whereas MERT cows showed greater (*p* < 0.001) mean rectal and vaginal temperatures in S than in W.

Rectal and vaginal temperatures showed a positive correlation (r = 0.90, *p* < 0.0001), but their correlations with OcularMin temperature were weak or absent, respectively. Cow-side THI showed a positive correlation with rectal temperature (r = 0.23, *p* < 0.01), and no significant correlation with vaginal temperature. The mean THI of the previous 60 days (THI60) showed positive correlation with OcularMin (r = 0.72, *p* < 0.0001), rectal (r = 0.42, *p* < 0.0001) and vaginal (r = 0.3, *p* < 0.01) temperatures. Based on these correlations, only OcularMin and rectal temperatures were considered for the multiple linear regression analysis. which considered the below model for the effects of Breed, THI (cow-side THI) and THI60:

$$\text{Temperature }=\upmu +\text{ Breed }+\text{ THI }+\text{ THI}60 +\text{ Breed}*\text{THI }+\text{ Breed}*\text{THI}60 +\text{ Breed}*\text{THI}*\text{THI}60 +\upvarepsilon$$

where μ is the overall intercept and ɛ is the random error or residual effect.

**Fig. 4:**
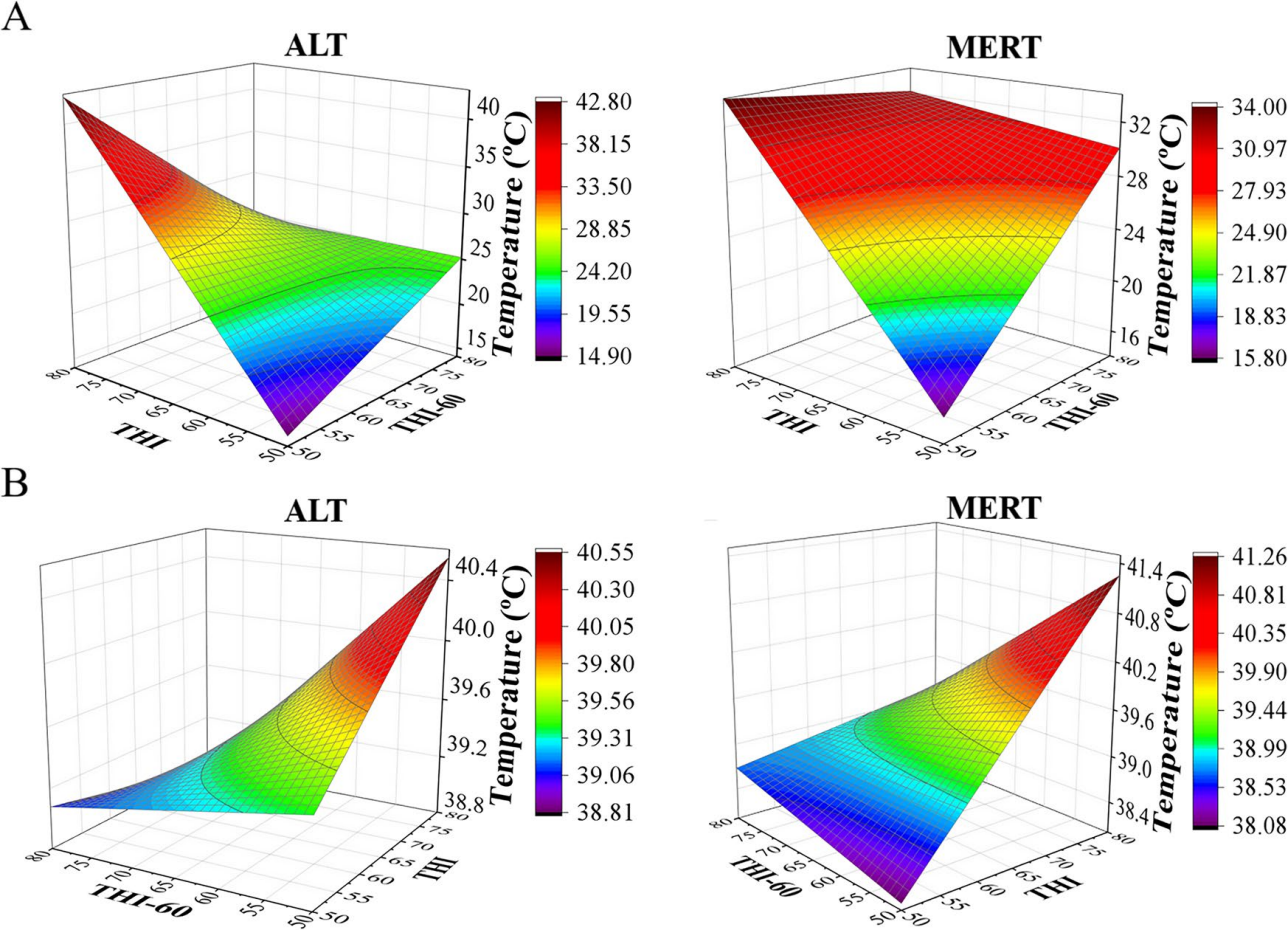
Graphical illustration of models for the interaction Breed*THI*THI60 in OcularMin **A** and rectal **B** temperatures in Alentejana (ALT) and Mertolenga (MERT) cows

As shown in Table 1 and illustrated in Fig. 4A, the OcularMin temperature model (*p* < 0.0001, r^2^ = 0.79, AICC = 444.78) included the significant effects of Breed, cow-side THI, THI60, and the interaction breed*THI*THI60 (*p* = 0.007). The OcularMin temperature increased with increasing THI and THI60. However, the increment per THI*THI60 unit was lower in ALT compared to MERT cows (0.014 °C difference between breeds). The model for rectal temperature illustrated in Fig. 4B (*p* < 0.0001, r^2^ = 0.65, AICC = 116.41) also included the significant effects of cow-side THI, THI60 and the interaction breed*THI*THI60 (*p* = 0.009). Rectal temperature increased with increasing THI and THI60, however the increment per THI*THI60 unit was lower in ALT than in MERT cows (0.0014 °C difference between breeds).

### Metabolic parameters

**Fig. 5:**
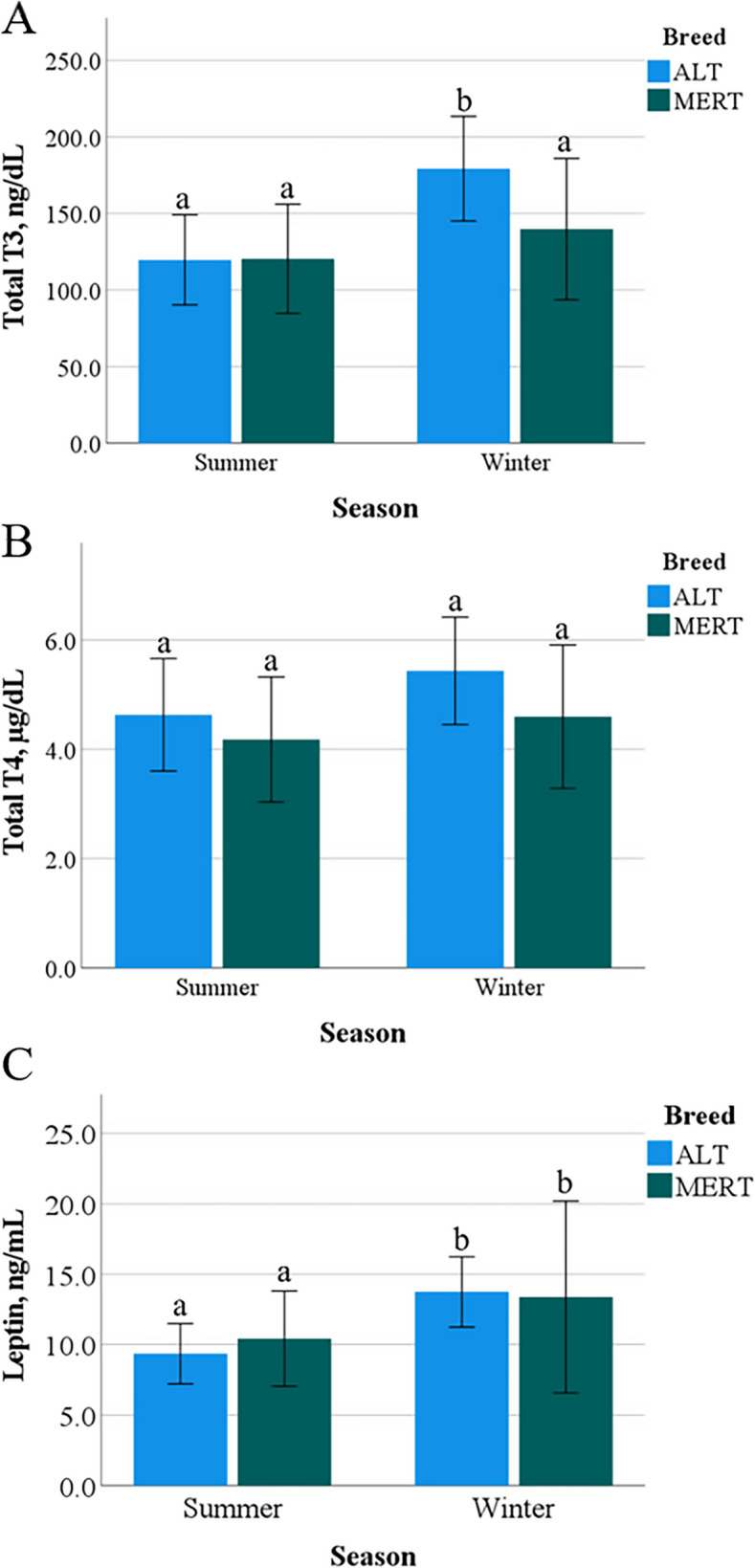
Total T3, T4 and leptin concentrations in Alentejana (ALT) and Mertolenga (MERT) cows; **A**—ab, *p* = 0.02; **B**—*p = 0.01; **p = 0.02; **C** – ab, *p* = 0.004

Body Condition Score was greater (*p* < 0.05) in ALT compared to MERT cows (6.7 ± 0.7 vs. 6.1 ± 0.5) and was not affected by Season or Breed*Season. Concentrations of BHB were similar in ALT and MERT cows (0.4 ± 0.2 mmol/L), without a Season or Breed*Season effect. Concentrations of T3 were affected by Breed (*p* < 0.05), Season (*p* < 0.0001) and Breed*Season (*p* < 0.05). MERT cows showed similar concentrations in S and W (121.5 ± 35.9 ng/dL vs. 139.1 ± 47.8 ng/dL), whereas ALT cows had lower concentrations in S than in W (119.7 ± 29.4 ng/dL vs. 179.3 ± 34.2 ng/dL) (Fig. 5A). Moreover, the decrease from W to S was more pronounced in ALT compared to MERT cows (34% vs. 13%). Concentrations of T4 were affected by Breed (ALT – 5.1 ± 1.1 µg/dL vs. MERT – 4.4 ± 1.3 µg/dL; *p* < 0.05) and Season (S – 4.4 ± 1.1 µg/dL vs. W – 5.0 ± 1.3 µg/dL; *p* < 0.05) (Fig. 5B).

Considering the evaluation of chronic HS on metabolic parameters, the GLM analysis of each metabolic parameter considered the following model:

$$\text{Metabolic parameter }=\upmu +\text{ Breed }+\text{ THI}60 +\text{ Breed}*\text{THI}60 +\upvarepsilon$$

where μ is the overall intercept and ɛ is the random error or residual effect.

Concentrations of T3 decreased when THI (r = −0.27, *p* < 0.05) and THI60 (r = −0.39, *p* < 0.001) increased, and this decrease was more pronounced (*p* < 0.05) in ALT than MERT cows. Concentrations of T4 also decreased (r = −0.29, *p* < 0.01) when THI60 increased in both breeds.

Leptin blood concentrations were only affected by Season (*p* < 0.01), being lower in S than in W (10.5 ± 3.0 ng/mL vs. 13.6 ± 5.2 ng/mL) (Fig. 5C). Leptin concentrations decreased when THI60 increased (r = −0.32, *p* < 0.01), at a rate of 0.15 ng/mL per THI60 unit. The decrease in leptin concentrations was correlated (r = 0.59, *p* < 0.0001) with the decrease in T3 concentrations, but not with that of T4 concentrations.

### Hair cortisol concentrations

**Fig. 6:**
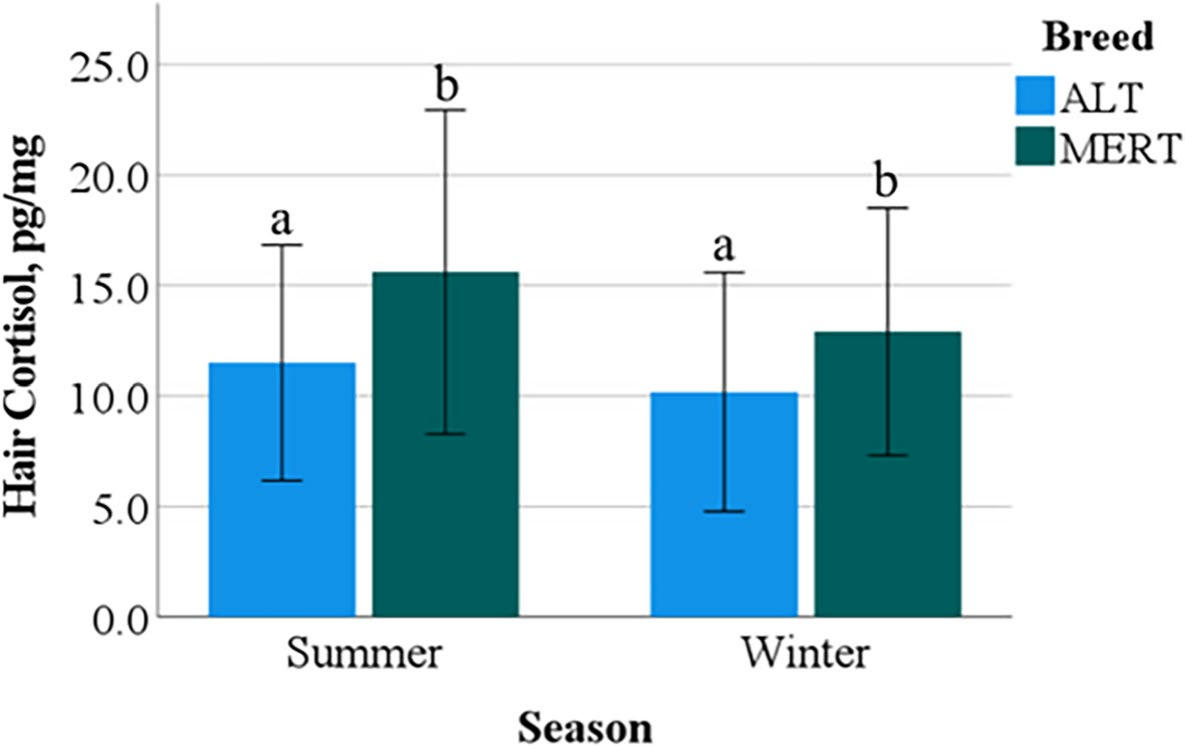
Interaction Breed*Season effect on hair cortisol concentrations of Alentejana (ALT) and Mertolenga (MERT) cows; ab, *p* = 0.01

The HCC were affected by Breed, as MERT cows had greater concentrations compared to ALT cows (14.2 ± 6.6 pg/mg vs. 10.8 ± 5.3 pg/mg, *p* < 0.05) (Fig. 6). However, the effects of Season and the interaction Breed*Season were not significant (ALT, 11.5 ± 5.3 pg/mg vs. 10.2 ± 5.4 pg/mg and MERT, 15.6 ± 7.3 pg/mg vs. 12.9 ± 5.6 pg/mg, in S vs. W, respectively) (Fig. 6). Hair cortisol concentrations were not significantly correlated with THI and THI60.

### Uterine health status

Uterine cytology PMN proportion was not affected by Breed (*p* = 0.5) or Breed*Season (*p* = 0.6) effects. In contrast, Season affected (*p* < 0.01) PMN proportion, as in both breeds the PMN proportion was lower in S compared to W (overall 0.5% vs. 2.2%). Both ALT and MERT cows had decreased uterine cytology PMN proportion with increasing THI60 (*p* < 0.001). However, considering the ≥ 5% uterine cytology PMN cutoff, only 5 (5.6%) cows exhibited endometritis. The biopsy-evaluated endometrial inflammatory infiltrate was not affected by Season (*p* = 0.9) but tended (*p* = 0.06) to be affected by Breed, as was evidenced in a greater proportion in ALT than MERT cows (71% vs. 46%). Neither the uterine cytology PMN proportion nor the presence of inflammatory infiltrate were correlated (*p* = 0.7) with HCC.

The subsequent calving to conception (overall: S—94.6 ± 24.1 and W—96.2 ± 33.0) and interval between calvings (overall: S—377.4 ± 25.4 and W—383.6 ± 34.7) were not affected by Breed, Season or Breed*Season effects.

## Discussion

The ALT and MERT native breeds evolved in an environmental scenario marked by long hot dry summers, which seems to have enhanced natural selection for genetic resistance to HS [38, 39], in one of the places on the globe most affected by climate change [1]. Interestingly, although reared in the same edaphoclimatic scenario, the present study showed that post-partum ALT and MERT cows developed breed-specific efficiencies and mechanisms in their thermoregulatory capacity to cope with HS.

In our study the main results regarding environmental temperature and THI during the two seasons are in accordance with [8]. In fact we prove that the meteorological data are similar between farms and herds in both conditions, with cow side THI and THI60 being higher in summer than the heat stress threshold [38, 39]

According to a previous study on cow thermodynamics [55], body temperature increases when HS goes beyond the limits of thermoregulation compensation mechanisms. These physiological mechanisms trigger body latent heat loss, mainly through vasodilation of superficial blood vessels [11, 14, 56]. Both ALT and MERT cows presented a linear increase in external body (ocular) temperature with an increase in THI, but the increment per THI unit was lower in ALT compared to MERT cows. However, a breed effect was observed in the response of internal (rectal and vaginal) temperatures to HS, as with increasing THI, MERT cows had a significant increase in internal temperatures, contrary to ALT cows. This evidenced that ALT cows appear to display more efficient thermoregulatory mechanisms for stabilizing body temperature under HS than MERT cows. As depicted by the significant interaction THI*THI60 for body temperatures, it is worth noting that the increase in body temperatures induced by THI is modulated by the THI of the previous 60 days, in the way that previous acclimation to high temperature attenuates the actual increase in body temperature. Apparently, this mechanism has evolved with different efficiency in ALT and MERT cows as denoted by the significant Breed*THI*THI60 interaction. Again, ALT cows display this attenuation mechanism more efficiently compared to MERT cows.

Modulation of thyroid hormones (T3 and T4) secretion to adjust the basal metabolism to environmental conditions [12] is a physiologic thermoregulatory mechanism to mitigate the impact of HS in body homeostasis [13, 14]. In the present study, both breeds exhibited a significant reduction in T3 serum concentrations in S compared to W. However, this reduction was more pronounced in ALT than in MERT cows (34% vs. 13%), which was associated with the lower increase in body temperatures observed in ALT compared to MERT cows. The level of reduction in T3 concentrations that was observed in ALT cows attained a level indicative of full acclimation (30%) [17] revealing a greater capacity for basal metabolism regulation. This can indicate that the process of deionization, through which the inactive T3 form turns active, is reduced under HS, and more efficiently in ALT than in MERT cows. According to [57] the mechanism of deionization, by which T4 is converted through the enzyme 5’-Deiodinase in the T3 active form, shows high plasticity, and can be organ specific. In accordance, although serum T4 concentrations were lower in S than in W in both breeds, they were greater in ALT than in MERT cows, denoting an accumulation of T4, due to less deionization to the T3 active form.

To evaluate if variations in T3 and T4 concentrations in S and W were due to adaptive mechanisms, the present study also evaluated metabolic parameters related with negative energy balance (BCS, BHB and leptin), which could be responsible for changes in thyroid hormone concentrations. It is established that the negative energy balance leads to decreased thyroid hormones secretion, thus reducing the metabolic rate and preserving metabolic reserves [19, 20, 58]. Due to shortage of pasture, both breeds received food supplementation in S. Results showed that BCS, although being constitutively greater in ALT than in MERT cows, was within the normal breed range, without change between seasons. Concentrations of BHB indicated that animals were not under negative energy balance. Thus, differences in T3 and T4 concentrations between S and W reflect adaptive mechanisms to acclimation to HS. In *Bos indicus* Sahiwal and Tharparkar breeds [59] a decrease in blood thyroid hormone concentrations during HS was reported, although more pronounced in the former than in the latter breed. In bulls of the well-known heat resistant Brahman breed, blood concentrations of T4 were increased in S, but those of T3 were not affected [15].

Leptin concentrations were lower in S than in W, which is a challenging result. Heat Stress is known to decrease food ingestion [60] and to increase leptin secretion [22], it therefore being expected that the decrease in T3 and T4 concentrations and associated basal metabolism in S would trigger an increase in leptin concentrations [21, 22]. This may represent a breed-developed mechanism to guarantee adequate food ingestion under HS, contributing to the resilience to the effects of high THI. The decrease in leptin concentrations was significantly correlated to that of T3 concentrations, suggesting an interaction between the two signaling pathways. Overall, this illustrates that cattle heat resilient breeds display adaptive metabolic mechanisms to cope with HS, but these show different pathways and efficiencies in different breeds. These adaptive mechanisms to HS are particularly efficient in the ALT breed, revealing phenotypic traits suitable for selection to resilience to HS. Studies in steers of heat-tolerant (Romosinuano) and heat-sensitive (Aberdeen-Angus) breeds also support the phenotype relevance to heat adaptability [61, 62].

Hair cortisol concentrations are currently the only method of assessing chronic stress, as all other matrices reflect stress of the previous minutes or days [63]. Cattle hair grows at an approximate rate of 0.6 mm per day [46] denoting that samples in the present study reflect cortisol concentrations from around the previous 60 days (2–3 cm near the skin). Several studies reported an increase in plasma cortisol concentrations associated to HS, not only in heat sensitive species/breeds, such as Saanen goats [64] and Holstein–Friesian cows [65], but also in heat resistant species/breeds, such as Murrah Buffaloes [24], Karan Fries cows [23] and *Bos indicus* breeds [15, 59]. In contrast, in the *Bos taurus* native breeds of this study, HCC were similar in S and W and were not correlated with THI of the previous 60 days which evidences that the thermoregulatory mechanisms put forward by these two breeds (ALT and MERT) under HS are independent of cortisol secretion, and HCC is not a reliable biomarker of HS. Additionally, this proves that in these two breeds the welfare is not severely impaired.

The post-partum uterine cytology PMN proportion was low in both breeds, as expected at 40 days [51]. However, it was lower in S compared to W, significantly decreasing with increasing THI60. These results may indicate that chronically increased THI leads to a lower PMN chemiotactic activity into the uterine lumen, potentially affecting uterine defense mechanisms. Although this lower post-partum uterine cytology PMN proportion in S was not responsible for an increase in uterine disease in ALT and MERT cows, this may predispose to uterine infection in less heat-resilient breeds. However, contrary to studies where an increase in uterine cytology PMN proportion was associated to an increase in cortisol secretion [66], results herein in ALT and MERT cows evidenced a mechanism independent of cortisol secretion. Although the uterine cytology and gynecological results showed an involuted uterus at 40 DPP, the biopsy evaluation still evidenced a high proportion of cows with significant inflammatory infiltrate in the deeper layers of the endometrium, both in S and W. This denotes an active restoration of endometrial homeostasis only ascertained at the histologic level, which might be helpful for uterine health. Indeed, all cows became pregnant within approximately 90 DPP, which is consistent with the high fertility of both breeds (> 90%).

## Conclusion

In conclusion, this study identified chronic stress phenotypic markers of beneficial thermoregulatory mechanisms of heat resilient cattle breeds, relevant for HS-resistance selection programs.

Results indicate that although reared in the same climatic scenario, ALT and MERT cows developed different strategies and efficiencies in the thermoregulatory mechanisms that render them highly resilient to chronic seasonal HS in the traditional continuous pasture system.

Ocular temperature was the body temperature more significantly related to THI, representing a valuable phenotypic biomarker of HS.

In their metabolic response to HS, both breeds exhibited a significant decrease in circulating T3 and T4 thyroid hormone concentrations, an adaptive mechanism to decrease the metabolic rate and endogenous heat production. A HS induced decrease in leptin secretion suggested an additional mechanism to stimulate food intake and maintain BCS and fertility, contributing to resilience towards high THI. Differences between the two breeds in the efficiency of the decrease of basal metabolism were associated with efficiency in maintaining external and internal body temperatures under increasing THI.

Chronic HS did not affect HCC in these breeds, revealing that the thermoregulatory mechanisms induced by HS are independent of cortisol secretion.

The endometrial cytology PMN proportion at 40 DPP, although low in both breeds, was reduced with increasing THI, denoting a negative effect of HS in the postpartum influx of PMN to the uterus, independent of cortisol secretion.

## Supplementary Information

Supplementary Material 1

## Data Availability

The datasets generated and/or analysed during the current study are not publicly available as they are the property of the collaborating associations, but are available from the corresponding author on reasonable request.

## Abbreviations

ALT: Alentejana breed
BCS: Body Condition Score
BHB: Beta-hydroxybutyrate
DPP: Days Postpartum
HS: Heat stress
HCC: Hair Cortisol Concentrations
IPMA: Portuguese Institute for Sea and Atmosphere, I. P.
MERT: Mertolenga breed
NEFA: Non-Esterified Fatty Acids
OcularMax: Maximal Ocular Temperature
OcularMean: Mean Ocular Temperature
OcularMin: Minimal Ocular Temperature
PMN: Polymorphonuclear neutrophils
S: Summer
THI60: Temperature-Humidity-Index of the previous 60 days
T3: Triiodothyronine
T4: Thyroxine
W: Winter
