# Supplementary material for: Effects of seasonal chronic heat stress on body thermoregulation, cortisol release and uterine health in postpartum native Alentejana and Mertolenga beef cattle
Source: BMC Vet Res. 2025 Jun 5;21:404. doi: 10.1186/s12917-025-04810-z (PMC12139315; doi:10.1186/s12917-025-04810-z)
Supplement: Supplementary file 1 — Supplementary Material 1 [file 12917_2025_4810_MOESM1_ESM.docx]

**Supplementary material**

**Effects of seasonal chronic heat stress on body temperatures, cortisol release, metabolic parameters and uterine health in native thermo-resilient postpartum beef cattle**

Luís G. Capela^1,2,3,4^, Inês C. Leites ^2,3^, Luísa M. Mateus^2,3^, Ricardo P. Romão^4^, Rosa MLN. Pereira ^2,3,4*^, Luís Lopes-da-Costa^2,3*^

Table 1. A- Floral composition of natural pastures; B - chemical analysis of hay in farms A and B.

| A | Specie |
| --- | --- |
|  | Trifolium subterraneum |
|  | Trifolium repens |
|  | Lolium perenne |
|  | Medicago polimorfa |
|  | Paspalum paspalodes |
|  | Avena fatua |
|  | Bomus arvensis |
|  | Lolium perenne |
|  | Medicago polimorfa |
|  | Ornithopus sativus |
|  | Poa pratensis |
|  | Festuca arundinacea |

| B | Parameter | Farm A | Farm B | Assay method |
| --- | --- | --- | --- | --- |
|  | Dry matter (%) | 95.6 | 91.5 | LIPP071 (Ed. 3) |
|  | Ash (%DM) | 4.8 | 7.6 | LIPP107 (ED. 1) |
|  | Crude protein (%DM) | 5.62 | 5.95 |  |
|  | NDF (%DM) | 75.4 | 56.3 |  |
|  | ADF (%DM) | 49.0 | 38.2 |  |
|  | Crude fiber (%DM) | 38.9 | 30.1 |  |

Table 2. Final GLM models used in the statistical analysis.

|  | | | | **Fixed effect p-value** | | | | | | | |
| --- | --- | --- | --- | --- | --- | --- | --- | --- | --- | --- | --- |
| **Model** | **Dependent Variable** | **Model r2** | **Model**  **p-value** | **Breed** | | | **Season** | | | **Breed*season** | |
| y = breed season breed*season | OcularMin | 0.49 | <0.0001 | 0.37 | | | <0.0001 | | | 0.265 | |
|  | OcularMax | 0.32 | <0.0001 | 0.578 | | | <0.0001 | | | 0.434 | |
|  | Vaginal | 0.23 | <0.0001 | 0.017 | | | 0.001 | | | 0.005 | |
|  | Rectal | 0.50 | <0.0001 | 0.002 | | | 0.001 | | | <0.001 | |
|  | BCS | 0.09 | 0.04 | 0.017 | | | 0.63 | | | 0.20 | |
|  | T4 | 0.13 | 0.009 | 0.005 | | | 0.0001 | | | 0.40 | |
|  | T3 | 0.27 | <0.0001 | 0.027 | | | <0.0001 | | | 0.016 | |
|  | BHB | 0.05 | 0.20 | 0.156 | | | 0.123 | | | 0.98 | |
|  | Leptin | 0.16 | 0.005 | 0.717 | | | 0.0003 | | | 0.303 | |
|  | Cortisol | 0.11 | 0.03 | 0.014 | | | 0.143 | | | 0.611 | |
|  | PMN# | NA^1^ | NA^1^ | 0.512 | | | 0.003 | | | 0.557 | |
|  | BCS | 0.09 | 0.04 | 0.017 | | | 0.625 | | | 0.202 | |
| **Model** | **Dependent variable** | **Model r2** | **Model**  **p-value** | **Breed** | **THI** | **THI60** | | **Breed*THI** | **Breed*THI60** | | **Breed*THI*THI60** |
| Y=breed THI60 Breed*THI60 | T4 | 0.14 | 0.006 | 0.336 | - | 0.012 | | - | 0.532 | | - |
|  | T3 | 0.25 | <0.0001 | 0.005 | - | 0.0001 | | - | 0.01 | | - |
|  | BHB | 0.05 | 0.235 | 0.958 | - | 0.168 | | - | 0.881 | | - |
|  | Leptin | 0.12 | 0.02 | 0.345 | - | 0.003 | | - | 0.303 | | - |
|  | Cortisol | 0.09 | 0.05 | 0.907 | - | 0.204 | | - | 0.805 | | - |
|  | PMN# | NA^2^ | NA^2^ | 0.311 | - | 0.0001 | | - | 0.271 | | - |
| Y=breed THI THI60 breed*THI breed *THI*THI60 | OcularMin | 0.79 | <0.0001 | 0.447 | <0.0001 | 0.0005 | | 0.275 | 0.551 | | 0.0007 |
|  | Rectal | 0.65 | <0.0001 | 0.221 | 0.054 | 0.003 | | 0.421 | 0.276 | | 0.0009 |

^1^Not applicable, the model goodness fit Pearson chi-square/DF is 1.02.

^2^Not applicable, the model goodness fit Pearson chi-square/DF is 1.002.

#Percentage of polymorphonuclear neutrophils present in uterine cytology
